# Melatonin Application in Assisted Reproductive Technology: A Systematic Review and Meta-Analysis of Randomized Trials

**DOI:** 10.3389/fendo.2020.00160

**Published:** 2020-03-27

**Authors:** Kai-Lun Hu, Xiaohang Ye, Siwen Wang, Dan Zhang

**Affiliations:** ^1^Key Laboratory of Reproductive Genetics, Ministry of Education, Department of Reproductive Endocrinology, Women's Hospital, Zhejiang University School of Medicine, Hangzhou, China; ^2^Key Laboratory of Women's Reproductive Health of Zhejiang Province, Hangzhou, China

**Keywords:** melatonin, assisted reproductive technology, randomized trial, *in vitro* fertilization, systematic review and meta-analysis

## Abstract

**Objective:** To study whether melatonin treatment can increase clinical pregnancy rate and live birth rate in assisted reproductive technology (ART) cycles.

**Methods:** Literature searches were conducted to retrieve randomized trials that reported the effect of melatonin treatment on ART outcomes. Databases searched included PubMed, EMBASE, Cochrane Library, Web of Science, and Google Scholar.

**Results:** Ten studies matched the inclusion criteria. Clinical pregnancy was reported in all of the included studies and live birth was reported in three studies. Melatonin treatment significantly increased the clinical pregnancy rate [OR = 1.43 (1.11, 1.86), power = 0.98, 10 RCTs, low-quality evidence] but not the live birth rate [OR = 1.38 (0.78, 2.46), power = 0.34, 3 RCTs, low-quality evidence]. Melatonin treatment increased the number of oocyte collected [SMD = 0.34 (0.01, 0.67), 7 RCTs, low-quality evidence], the number of maturated oocyte [SMD = 0.56 (0.27, 0.85), 7 RCTs, low-quality evidence], and the number of good quality embryo [MD = 0.36 (0.18, 0.55), 3 RCTs, low-quality evidence]. Melatonin treatment significantly increased the biochemical pregnancy rate [OR = 1.65 (1.14, 2.38), 6 RCTs, low-quality evidence] and had no significant effect on the miscarriage rate [OR = 1.28 (0.65, 2.51), 5 RCTs, low-quality evidence].

**Conclusion:** Melatonin treatment significantly increases the clinical pregnancy rate but not live birth rate in ART cycles. Melatonin treatment also increases the number of oocyte collected, maturated oocyte, and good quality embryo. No clear evidence suggested that melatonin treatment increased the adverse events in ART cycles. The actual findings may be compromised due to the wide heterogeneity of the included IVF patients, from PCOS to low ovarian reserve.

## Introduction

Infertility is defined as the inability to conceive for at least 1 year (1). Infertility is a common condition affecting 10–20% of women at the reproductive age across different countries (2, 3). Assisted reproductive technology (ART) has allowed millions of infertile couples in the world to conceive successfully since 1978 (4). Although substantial advance has been made in the past decades, the chance of achieving a live birth through ART is not high (5, 6). Several strategies aiming to increase the live birth rate are currently being used, such as endometrial scratching (7), assisted hatching of human embryos (8), the addition of drugs to improve successful rate (9, 10), etc.

The successful rate of ART cycles can be affected by several internal as well as external factors (11, 12). Oxidative stress is a state characterized by an imbalance between pro-oxidant molecules and antioxidant defenses and it plays a vital role in the pathogenesis of female infertility (13, 14). Oxidative stress causes toxic effects on oocyte maturation and is considered one of the causes of poor oocyte quality (15). Increased oxidative stress can lead to reduced oocyte maturation rate and fertilization rate, which will result in a reduced possibility of full-term pregnancy (15). Melatonin is the main hormone derived from the pineal gland. Other extrapineal organs, like the gastrointestinal tract and female ovary, can also secret this hormone (16). Melatonin has been reported to regulate several physiological processes, including circadian rhythms, endoplasmic reticulum stress response, apoptosis and autophagy, and mitochondrial homeostasis (16, 17). Melatonin and its metabolites also protect cells from oxidative stress by acting as a free radical scavenger (17).

In the last decade, many studies, including randomized trials, have reported the application of melatonin in ART cycles. However, the sample size is small in most of these studies and the evidence generated is of low quality. Additionally, the results in these studies are controversial and no definite conclusion has been made currently. In this systematic review, we aim to perform an in-depth overview to evaluate the effects of melatonin application in ART cycles.

## Methods

### Inclusion Criteria

We included randomized trial studies that investigated melatonin application in ART cycles, including *in vivo* treatment and *in vitro* application for oocyte or embryo culture. Studies not written in English were excluded. Reviews, conference abstracts, case report studies, and study protocols were also excluded.

### Literature Search

Two authors (K-LH and XY) independently searched the database of PubMed, EMBASE, Cochrane Library, Web of Science, and Google Scholar from January 1978 to November 2019. The PICO search method was used to collect related literature. Patients were those who underwent ART cycles, including intrauterine insemination (IUI), *in vitro* fertilization (IVF) and/or Intra-Cytoplasmic Sperm Injection (ICSI) and subsequent embryo transfer (ET). Interventions included melatonin or its analog treatment with or without any adjuvant treatment. The comparators were treatments without melatonin or placebo. Outcomes included clinical pregnancy, live birth rate, oocyte and embryo quality, and miscarriage. And the study design was randomized trials. The key search terms included but not limited to “melatonin,” “assisted reproductive technology,” “*in vitro* fertilization,” “live birth,” “oocyte quality,” “randomized trials.” The detailed search terms and methods could be seen in Supplemental Table 1.

### Study Selection

Two authors (K-LH and XY) independently scrutinized all of the titles and abstracts according to the predefined inclusion criteria. The full manuscripts of the studies were obtained if the titles and abstracts were considered to be relevant for inclusion. Any disagreement between the two authors was resolved by a third review author (DZ). References of all included studies judgments to identify relevant articles not captured by the electronic searches.

### Data Extraction

Two authors (K-LH and XY) independently extracted data from included trials. Any disagreements were solved by consulting another author (DZ). In cases we identified a study with multiple publications, the main trial report was used as the reference and additional details were supplemented from other papers. The data extracted from the eligible studies included the sample size, publication year, time frame, country, inclusion and exclusion criteria, diagnosis of participants, the protocol for ART, the definition of outcomes, and the data of outcomes.

### Study Quality Assessment and Publication Bias

Two reviewers (K-LH and SW) independently conducted the quality assessment of the included studies. To evaluate the risk of bias, we followed the Cochrane Collaboration's criteria (version 5.1.0, Available from www.cochrane-handbook.org) for judging the risk of bias and the studies were classified as being of low, high, or unclear risk of bias. Funnel plot was used to assess the publication bias.

### Statistical Analysis

The Review Manager version 5 was used to merge and analyze the extracted data. Forest plots were created for each outcome. The results were combined for meta-analysis using the Mantel/Haenszel model. A fixed-effect model was used where no statistically significant heterogeneity is present (*I*^2^ <50%). When substantial heterogeneity was observed (*I*^2^ > 50%), we would address it by rechecking data and excluding studies with a high risk of bias for sensitivity analysis. If substantial heterogeneity persisted, a random-effect model was used. The discontinuous results were shown by odds ratio (OR) with a 95% confidence interval (CI). The continuous results were shown by the difference in means (MD) with 95% CI for a fixed-effect model or standard mean difference (SMD) with 95% CI for a random-effect model. Statistical significance is set at a *P* level of 0.05. The statistical power of the meta-analysis was conducted for the main outcomes.

### Outcomes and Additional Analysis

The primary outcomes included clinical pregnancy rate and live birth rate. Secondary outcomes included oocyte retrieval number, the number of the maturated oocyte (MII), the number of the top quality embryo, biochemical pregnancy rate, miscarriage rate, and adverse event. Subgroup analysis was conducted for different protocols of melatonin application and only clinical pregnancy rate was addressed. In cases that there were multiple doses of melatonin treatment in a single study, the data of all the treatment groups were first merged and then was considered as one intervention group.

## Results

### Characteristics of the Included Studies

**Figure 1:**
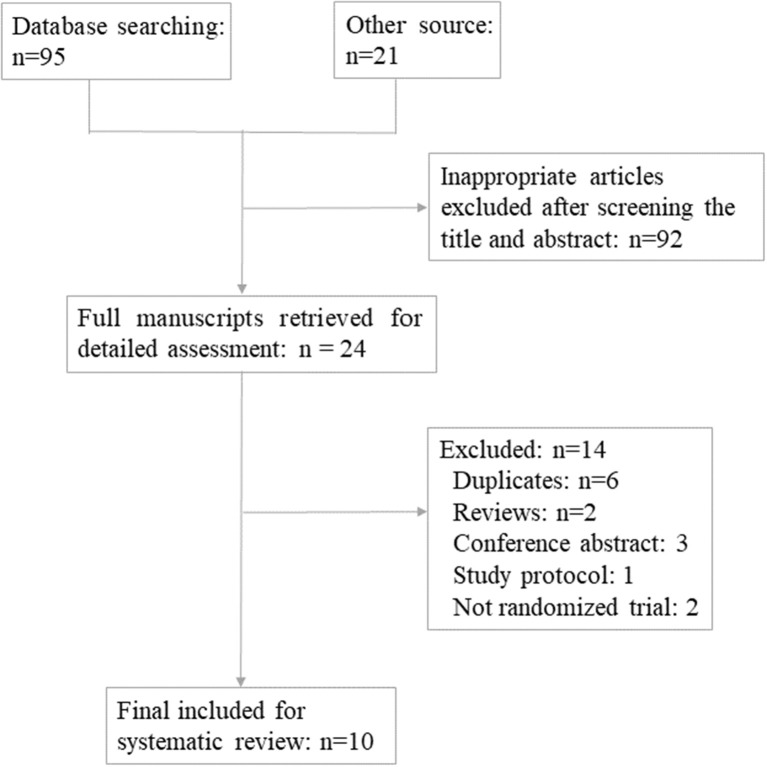
Flow chart for included studies.

The PRISMA flow diagram of the review process was presented in Figure 1. A total of 10 randomized trials were included for analysis (18–27). Characteristics of the included studies were presented in Tables 1, 2. The included studies varied in publication date from 2010 to 2019. A total of 1,203 participants were included for analysis. Six hundred and forty five were allocated to the melatonin treatment group and 558 were allocated to the control group. The funnel plot showed no publication bias for the included studies (Supplemental Figure 1). The qualitative analysis of the included studies could be found in Supplemental Figure 2. Each risk of bias item presented as percentages across all included studies could be found in Supplemental Figure 3. Most studies were at high risk of bias and only two studies were of good quality (22, 24). From these included studies, sample sizes varied from 30 women to 331 women. Nine were *in-vivo* studies and 1 was the *in-vitro* application of melatonin. Three studies focused on women with polycystic ovarian syndrome (PCOS); the patients in 3 studies were none special; the other 4 studies focused on women with unexplained infertility, diminished ovarian reserve, poor oocyte quality, and sleep disturbances, respectively. One study included women undergoing IUI and 9 studies focused on women undergoing *in vitro* fertilization (IVF) and/or Intra-Cytoplasmic Sperm Injection ICSI and subsequent embryo transfer ET. The dose of 3 mg melatonin was most commonly used in these studies. One study used 10 μmol/l in the culture medium for embryo culture (26). One study used 2/4/8 mg melatonin for intervention in three parallel groups (24). Three studies used 3 mg melatonin together with myo-inositol and folic acid (18, 20, 21). Other detailed characteristics of these studies could be found in Tables 1, 2.

**Table 1:** Characteristics of included studies.

**References**	**Conflict of interest**	**Ethical approval**	**Time frame**	**Country**	**Diagnosis of participants**	**Sample size**	**Inclusion criteria**	**Exclusion criteria**
Tunon et al. (18)	NR	NR	NR	Spain	NS	120	Age 18-41 years, BMI from 18 to 29 kg/m2, and normal ovulatory cycle of 24-35 days	Azoospermia; abnormalities of the reproductive system; potential causes of ovulatory dysfunction; hypersensitivity to gonadotropin
Jahromi et al. (19)	None	Yes	2014-2015	Iran	Diminished ovarian reserve	66	The first ART cycle, normal male factor and uterine cavity, and 2 of the criteria: (1) bilateral AFC ≤ 6; (2) AMH ≤ 1; 3) basal FSH ≥ 10	Declined to participate, poor compliance, or the ovaries showed poor responses to gonadotropins
Pacchiarotti et al. (20)	Yes^a^	Yes	2009-2011	Italy	PCOS	331	Basal FSH < 12 IU/L; Rotterdam criteria for PCOS; BMI 20–26 kg/m2, and first IVF treatment	Declined to participate; tubal, uterine, genetics and male causes of infertility
Rizzo et al. (21)	NR	Yes	NR	Italy	Low oocyte quality history	65	Age 35–42; low oocyte quality detected in the previous IVF cycles	NR
Mokhtari et al. (22)	None	Yes	2017	Iran	PCOS	198	Aged 20–40; normal sperm available; normal hysterosalpingography; Rotterdam criteria for PCOS; no endocrine diseases; no hormonal drugs use within the past three months	Porr ovarian response; ovarian hyperstimulation syndrome; no history of treatment for infertility
Eryilmaz et al. (23)	None	Yes	2010	Turkey	Sleep disturbances	60	Disturbed sleep status; unexplained infertility; without ovulatory and hysterosalpingography or laparoscopy problem	Chronic drug usage, history of IVF failure, hypertension, DM, uterine myoma, ovarian cyst, and smoking
Fernando et al. (24)	None	Yes	2014-2016	Australia	NS	160	First IVF/ICSI cycle; aged 18–45; BMI 18–35 kg/m^2^	Untreated endometriosis, uterine malformations, AD, other adjuvant therapies, malignancy, PGT
Batioglu et al. (25)	None	NR	NR	Turkey	NS	85	Primary infertility; age 20–40; regular menstrual cycles; no hormonal therapy for the last 3 months; no systemic illness	Serious endometriosis; azoospermia; hypogonatropic hypogonadism; FSH >13
Kim et al. (26)	None	Yes	2004–2008	Korea	PCOS	111	Rotterdam criteria for PCOS; poor response to clomiphene citrate; not conceived after several cycles of ovulation induction	NR
Espino et al. (27)	None	Yes	NR	Spain	UI	30	UI, normospermic, normal ovulation	<18 years, active smokers, concurrently using other adjuvant therapies

*NR, not reported; NS, no special; ET, embryo transfer; IVF, in vitro fertilization; PCOS, polycystic ovarian syndrome; BMI, body mass index; DM, diabetes mellitus; PGT, preimplantation genetic screening; AD, autoimmune disease; UI, unexplained infertility*.

a *One author is the employee at Pharma SRL*.

**Table 2:** Protocol and outcomes definition in the included studies.

**References**	**ART type**	**Down-regulation protocol**	**ET protocol**	**Intervention protocol**	**Intervention time**	**Control**	**Biochemical pregnancy**	**Clinical pregnancy**	**Live birth**	**Miscarriage**
Tunon et al. (18)	ICSI-ET	GnRH antagonist or GnRH agonist	Day 2 to 5; ET number 1–3	2 doses of 0.975 mg melatonin, 2 g myo-inositol, 200 μg FA, and 27.5 μg selenium	For at least 2 months before the ovarian puncture	NR	HCG > 50 mU/mL 12 to 15 days after ET	Gestational sacs and fetal cardiac activity under transvaginal ultrasonography	NR	NR
Jahromi et al. (19)	IVF-ET	GnRH agonist	Day 3, ET number NR	3 mg melatonin every night	From the day 5 prior to COS up to ovum pickup	Placebo	Elevation in serum β-hCG levels 16 days after ET	Embryo with cardiac activity	NR	NR
Pacchiarotti et al. (20)	ICSI-ET	GnRH agonist	Day 2, ET number NR	Myo-inositol (4000 mg), FA (400 mcg) and melatonin (3 mg)	From the first day of the cycle until 14 days after ET	Myo-inositol (4000 mg) and FA (400 mcg)	NR	Presence of a gestational sac on ultrasonography	NR	NR
Rizzo et al. (21)	IVF-ET	GnRH agonist	NR	2 g myo-inositol twice with 200 mg FA and 3 mg melatonin	From the day of GnRH agonist treatment	2 g myo-inositol twice with 200 mg FA	Increase in β-hCG	Embryo with cardiac activity	NR	Loss of the pregnancy at 5-12 weeks of gestation.
Mokhtari et al. (22)	IUI	None	NA	3 mg melatonin	From day 3 to the triggering day	Placebo	β-hCG test	NR	NR	NR
Eryilmaz et al. (23)	IVF-ET	GnRH agonist	Day 3, ET number 1-3	3 mg melatonin	From the 3rd to the 5th day until the triggering day	None	β-hCG ≥ 20 IU/L on the 12th day after ET	Presence of a gestational sac on ultrasonography	NR	NR
Fernando et al. (24)	IVF or ICSI-ET	GnRH antagonist	Day 3 or 5, ET number NR	2/4/8 mg melatonin	From day 2 until the day before oocyte retrieval	Placebo	NR	A live intrauterine pregnancy on transvaginal ultrasound	NR	NR
Batioglu et al. (25)	IVF-ET	GnRH agonist	NR	3 mg melatonin	NR	None	NR	NR	NR	NR
Kim et al. (26)	IVM-IVF-ET	NA	ET day NR, ET number 2-3	10 umol/l in the culture medium	24–48 h	None	NR	Positive β-hCG with an intrauterine pregnancy	NR	NR
Espino et al. (27)	ICSI-ET	GnRH antagonist	Day 2 or 3, ET number 1	3/6 mg melatonin	from the first appointment to COS until ovum pickup	None	NR	NR	NR	NR

*NR, not reported; NA, not applicable; ET, embryo transfer; IVF, in vitro fertilization; ICSI, Intra-Cytoplasmic Sperm Injection; IUI, intrauterine insemination; COS, control ovarian stimulation; FA, folic acid*.

### Primary Outcome

#### Clinical Pregnancy per Allocated Woman

**Figure 2:**
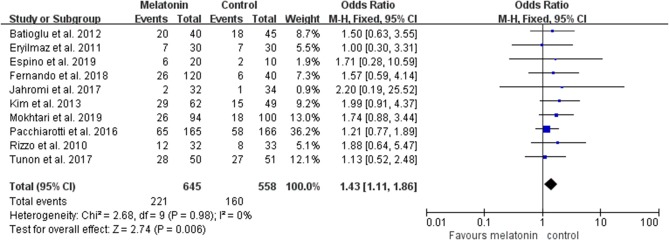
Meta-analysis of studies reporting the rate of clinical pregnancy. Meta-analysis of the data from all 10 of the included studies that reported clinical pregnancy as an outcome showed that women treated with melatonin had a higher chance of achieving clinical pregnancy from ART when compared with the controls.

Ten studies reported the effect of melatonin on clinical pregnancy (Figure 2). Meta-analysis suggested that melatonin treatment significantly increased clinical pregnancy rate [OR = 1.43 (1.11, 1.86), *P* < 0.01, power = 0.98]. The studies included for meta-analysis had low heterogeneity with an *I*^2^ value of 0%.

#### Live Birth per Allocated Woman

**Figure 3:**
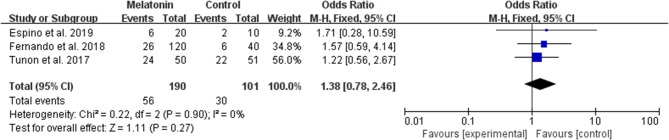
Meta-analysis of studies reporting the live birth rate. Meta-analysis of the data from 3 of the included studies that reported live birth as an outcome showed that women treated with melatonin did not have a significantly increased rate of live birth from ART.

Three studies reported the effect of melatonin on live birth (Figure 3). Meta-analysis suggested that melatonin treatment did not increase the live birth rate [OR = 1.38 (0.78, 2.46), *P* > 0.05, power = 0.34]. The studies included for meta-analysis had low heterogeneity with an *I*^2^ value of 0%.

### Secondary Outcome

#### The Average Number of Oocyte Retrieved per Allocated Woman

**Figure 4:**
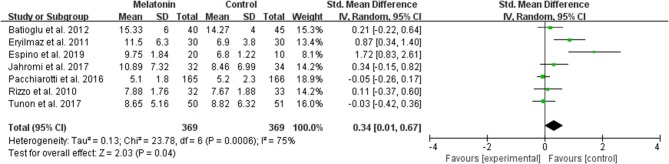
Meta-analysis of studies reporting the number of oocyte retrieved. Meta-analysis of the data from 7 of the included studies that reported the number of oocyte retrieved showed that women treated with melatonin had a significantly increased number of oocyte retrieved from ART.

The data of the number of oocytes collected were available to be extracted and synthesized in 7 studies (Figure 4). Meta-analysis suggested that melatonin treatment significantly increased the number of oocyte collected [SMD = 0.34 (0.01, 0.67), *P* < 0.05, random-effect].

#### The Average Number of Maturated Oocyte per Allocated Woman

**Figure 5:**
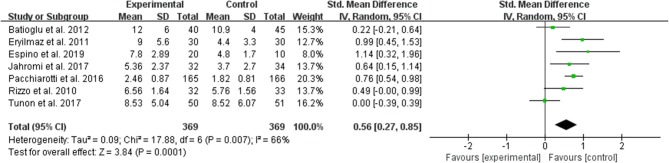
Meta-analysis of studies reporting the number of the maturated oocyte. Meta-analysis of the data from 7 of the included studies that reported the number of maturated oocyte showed that women treated with melatonin had a significantly increased number of the maturated oocyte from ART.

The data of comparison of the number of maturated oocyte were available to be extracted and synthesized in 7 studies (Figure 5). Meta-analysis suggested that melatonin treatment significantly increased the number of maturated oocyte [SMD = 0.56 (0.27, 0.85), *P* = 0.0001, random-effect].

#### The Average Number of Good Quality Embryo per Allocated Woman

**Figure 6:**
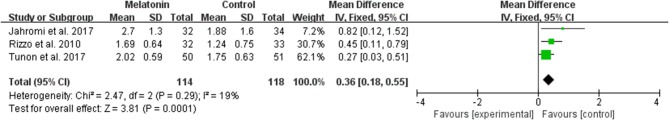
Meta-analysis of studies reporting the number of top quality embryo. Meta-analysis of the data from 3 of the included studies that reported the number of top quality embryo showed that women treated with melatonin had a significantly increased number of the top quality embryo from ART.

The data of comparison of the number of good quality embryos were available to be extracted and synthesized in 3 studies (Figure 6). Meta-analysis suggested that melatonin treatment significantly increased the number of good quality embryo [MD = 0.36 (0.18, 0.55), *P* = 0.0001]. The studies included for meta-analysis had low heterogeneity with an *I*^2^ value of 19%.

#### Biochemical Pregnancy per Allocated Woman

**Figure 7:**
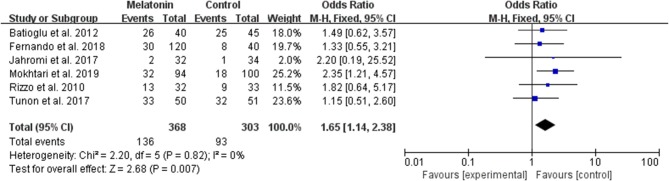
Meta-analysis of studies reporting the biochemical pregnancy rate. Meta-analysis of the data from 6 of the included studies that reported biochemical pregnancy as an outcome showed that women treated with melatonin had a higher chance of achieving biochemical pregnancy from ART when compared with the controls.

Six studies reported the effect of melatonin on biochemical pregnancy (Figure 7). Meta-analysis suggested that melatonin treatment significantly increased the biochemical pregnancy rate [OR = 1.65 (1.14, 2.38), *P* < 0.01]. The studies included for meta-analysis had low heterogeneity with an *I*^2^ value of 0%.

#### Miscarriage per Allocated Woman

**Figure 8:**
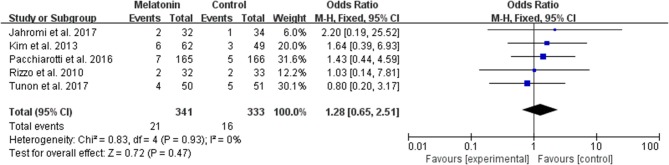
Meta-analysis of studies reporting the miscarriage rate. Meta-analysis of the data from 5 of the included studies that reported miscarriage as an outcome showed that women treated with melatonin did not have a significantly increased rate of miscarriage from ART when compared with the controls.

The data of the rate of miscarriage were available to be extracted and synthesized in 5 studies (Figure 8). Meta-analysis suggested that melatonin treatment significantly had no significant effect on the miscarriage rate [OR = 1.28 (0.65, 2.51), *P* > 0.05]. The studies included for meta-analysis had low heterogeneity with an *I*^2^ value of 0%.

#### Adverse Events

One study reported that one woman treated with melatonin had a term live birth of a baby with an absent right kidney. Two patients were diagnosed with pre-eclampsia. One patient was diagnosed with placenta previa (24). Other potential adverse events, like ectopic pregnancy and ovarian hyperstimulation syndrome, were not different between the melatonin treatment group and the control group, or not reported.

### Subgroup Analysis

#### Clinical Pregnancy per Allocated Woman in IVF/ICSI-ET Cycles

Nine studies reported the effect of melatonin on clinical pregnancy in IVF/ICSI-ET cycles (18–21, 23–27). Meta-analysis suggested that melatonin treatment significantly increased clinical pregnancy rate [OR = 1.39 (1.05, 1.84), *P* < 0.05]. The studies included for meta-analysis had low heterogeneity with an *I*^2^ value of 0%.

#### Clinical Pregnancy per Allocated Woman in *in-vivo* Studies

Nine *in-vivo* studies reported the effect of melatonin on clinical pregnancy (18–25, 27). Meta-analysis suggested that melatonin treatment significantly increased clinical pregnancy rate [OR = 1.38 (1.05, 1.81), *P* < 0.05]. The studies included for meta-analysis had low heterogeneity with an *I*^2^ value of 0%.

#### Clinical Pregnancy per Allocated Woman for Melatonin vs. Control Studies

Six *in-vivo* studies reported the effect of melatonin vs. control on clinical pregnancy (19, 22–25, 27). Meta-analysis suggested that melatonin treatment significantly increased clinical pregnancy rate [OR = 1.55 (1.02, 2.35), *P* < 0.05]. The studies included for meta-analysis had low heterogeneity with an *I*^2^ value of 0%.

#### Clinical Pregnancy per Allocated Woman for Myo-inositol Plus Melatonin vs. Myo-inositol Studies

Two *in-vivo* studies reported the effect of included plus melatonin vs. myo-inositol on clinical pregnancy (20, 21). Meta-analysis suggested insignificant difference between the two groups [OR = 1.29 (0.86, 1.95), *P* > 0.05]. The studies included for meta-analysis had low heterogeneity with an *I*^2^ value of 0%.

#### Clinical Pregnancy in Women With PCOS

Three studies reported the effect of melatonin treatment on clinical pregnancy in women with PCOS (20, 22, 26). Meta-analysis suggested a significant effect of melatonin treatment [OR = 1.45 (1.04, 2.03), *P* < 0.05]. The studies included for meta-analysis had low heterogeneity with an *I*^2^ value of 0%. Two *in-vivo* studies reported the effect of melatonin treatment on clinical pregnancy in women with PCOS (20, 22). Meta-analysis suggested an insignificant effect [OR = 1.35 (0.93, 1.96), *P* > 0.05]. The studies included for meta-analysis had low heterogeneity with an *I*^2^ value of 0%.

## Discussion

This systematic review includes 10 randomized control studies for quantitative analysis and the result indicates that melatonin treatment significantly increases the clinical pregnancy rate in ART cycles, and the effect is probably mediated by increasing the quality of oocytes and embryos. Melatonin treatment has no significant effect on the live birth rate and the result needs to be confirmed by future studies with a large sample size.

Melatonin has been reported to regulate several physiological processes, including circadian rhythms, endoplasmic reticulum stress response, apoptosis and autophagy, and mitochondrial homeostasis (16, 17). Melatonin and its metabolites also protect cells from oxidative stress by acting as a free radical scavenger that is able to deactivate a variety of reactive oxygen species (17, 28, 29). Accumulating studies indicate that increased oxidative stress in the peritoneal, serum, and follicular microenvironments can result in poor oocyte quality and compromise the reproductive potential of women (30–32). It is likely that reducing the reactive oxygen species in the microenvironment can protect the oocyte and the embryo from oxidative stress. Indeed, recent studies suggest that melatonin concentration in follicular fluid is associated with oocyte maturation rate and good quality embryo rate in women undergoing ART procedures (33). Additionally, melatonin treatment in females for 3 mg per day or higher doses can significantly increase the serum and follicle concentration of melatonin (22, 24, 27). Therefore, it is reasonable that melatonin treatment can increase oocyte and embryo quality and subsequent pregnancy outcomes. It should be noted that melatonin concentration dynamically changes within a day. In humans, melatonin secretion begins since nightfall, reaches a peak level in the middle of the night and decreases gradually during the second half of the night (34). Therefore, it is necessary to collect the samples at a specific time in all participants.

A previous prospective, longitudinal, cohort study indicates that myo-inositol plus melatonin treatment significantly improves oocyte quality in women who failed to conceive in previous *in vitro* fertilization cycles due to poor oocyte quality (35). Additionally, the number of maturated oocytes and the number top-quality embryos transferred were significantly higher than the previous IVF cycle (35). In agreement, one randomized trial including women with poor oocyte quality history shows that melatonin together with myo-inositol and folic acid treatment produces more maturated oocytes and top quality embryos than myo-inositol and folic acid treatment (21). However, the clinical pregnancy rate is not significantly increased in the melatonin together with myo-inositol and folic acid treatment group (21). The negative result of the clinical pregnancy rate in this study can be attributed to the small sample size. Consistent with these studies, our meta-analysis also suggests that melatonin treatment significantly increases the number of maturated oocytes and the number of good quality embryos. A previous meta-analysis shows that melatonin treatment does not significantly increase clinical pregnancy in women undergoing ART (36). This study includes only five randomized trials and the sample size is not large. It is necessary to update the data because recent randomized studies show improved pregnancy outcomes after melatonin treatment in ART cycles. Furthermore, our study also shows that the number of oocytes retrieved is significantly increased, although the significance is not detected most randomized trials. The application of melatonin in patients with PCOS is promising according to our study, and future studies addressing the role of melatonin in these patients will be interesting.

Our study shows that the miscarriage rate is not different between the melatonin treatment group and the control group. Several obstetric complications are reported in women receiving melatonin treatment (24). Although it does not necessarily mean that melatonin treatment increases the rate of obstetric complications because only one patient for each complication is reported, future studies with a large sample size should better report the data and provide robust evidence.

The main limitation of this study is the low quality of most studies included in the meta-analysis. Additionally, a limited number of cases are included in most individual studies and the data of live birth is reported in only 3 studies. Even we combined the data of these studies, we did not found the improvement of live birth rate after the treatment of melatonin. The negative result may result from the small sample size and future studies address the effect of melatonin in ART cycles should better report this outcome and provide more robust evidence. The wide heterogeneity of the included IVF patients, from PCOS to low ovarian reserve, may compromise the actual findings in this study.

## Conclusion

This systematic review suggests that melatonin treatment significantly increases the clinical pregnancy rate in ART cycles. Melatonin treatment also increases the number of oocyte collected, maturated oocyte, and good quality embryo. No clear evidence suggested that melatonin treatment increased the adverse events in ART cycles. Melatonin treatment has no significant effect on the live birth rate and the result needs to be confirmed by future studies with a large sample size. The actual findings may be compromised due to the wide heterogeneity of the included IVF patients, from PCOS to low ovarian reserve.

## Author Contributions

K-LH and XY reviewed the literature and extracted the data. K-LH and SW assessed the quality of included studies. K-LH designed the study, wrote the manuscript, and designed the figures and tables. DZ provided some key ideas for this manuscript. All authors participated in the discussion of analysis and interpretation of data in this article.

### Conflict of Interest

The authors declare that the research was conducted in the absence of any commercial or financial relationships that could be construed as a potential conflict of interest.

## Supplementary Material

Supplemental Figure 1 Funnel plot of the included studies.

Supplemental Figure 2 Risk of bias summary: review authors' judgments about each risk of bias item for each included study.

Supplemental Figure 3 Risk of bias graph: review authors' judgments about each risk of bias item presented as percentages across all included studies.

Supplemental Table 1 Search methods for included studies.

## References

[1] Gnoth C Godehardt E Frank-Herrmann P Friol K Tigges J Freundl G. Definition and prevalence of subfertility and infertility. Hum Reprod. (2005) 20:1144–7. 10.1093/humrep/deh870 15802321

[2] Thoma ME McLain AC Louis JF King RB Trumble AC Sundaram R. Prevalence of infertility in the United States as estimated by the current duration approach and a traditional constructed approach. Fertil Steril. (2013) 99:1324–31.e1321. 10.1016/j.fertnstert.2012.11.037 23290741 PMC3615032

[3] Zhou Z Zheng D Wu H Li R Xu S Kang Y. Epidemiology of infertility in China: a population-based study. BJOG. (2018) 125:432–41. 10.1111/1471-0528.14966 29030908

[4] Kupka MS Ferraretti AP de Mouzon J Erb K D'Hooghe T Castilla JA. Assisted reproductive technology in Europe, 2010: results generated from European registers by ESHREdagger. Hum Reprod. (2014) 29:2099–113. 10.1093/humrep/deu175 25069504

[5] Gunby J Bissonnette F Librach C Cowan L. Assisted reproductive technologies (ART) in Canada: 2007 results from the Canadian ART Register. Fertil Steril. (2011) 95:542–7.e541-10. 10.1016/j.fertnstert.2010.05.057 20656287

[6] Wade JJ MacLachlan V Kovacs G. The success rate of IVF has significantly improved over the last decade. Aust N Z J Obstet Gynaecol. (2015) 55:473–6. 10.1111/ajo.12356 26174052

[7] Vitagliano A Andrisani A Alviggi C Vitale SG Valenti G Sapia F. Endometrial scratching for infertile women undergoing a first embryo transfer: a systematic review and meta-analysis of published and unpublished data from randomized controlled trials. Fertil Steril. (2019) 111:734–46.e732. 10.1016/j.fertnstert.2018.12.008 30683590

[8] Martins WP Rocha IA Ferriani RA Nastri CO. Assisted hatching of human embryos: a systematic review and meta-analysis of randomized controlled trials. Hum Reprod Update. (2011) 17:438–53. 10.1093/humupd/dmr012 21474527

[9] Kolibianakis EM Venetis CA Diedrich K Tarlatzis BC Griesinger G. Addition of growth hormone to gonadotrophins in ovarian stimulation of poor responders treated by *in-vitro* fertilization: a systematic review and meta-analysis. Hum Reprod Update. (2009) 15:613–22. 10.1093/humupd/dmp026 19561136

[10] Showell MG Mackenzie-Proctor R Jordan V Hart RJ. Antioxidants for female subfertility. Cochrane Database Syst Rev. (2017) 7:Cd007807. 10.1002/14651858.CD007807.pub3 28752910 PMC6483341

[11] Mantikou E Youssef MA van Wely M van der Veen F Al-Inany HG Repping S. Embryo culture media and IVF/ICSI success rates: a systematic review. Hum Reprod Update. (2013) 19:210–20. 10.1093/humupd/dms061 23385469

[12] Tolunay HE Sukur YE Ozkavukcu S Seval MM Ates C Turksoy VA. Heavy metal and trace element concentrations in blood and follicular fluid affect ART outcome. Eur J Obstet Gynecol Reprod Biol. (2016) 198:73–7. 10.1016/j.ejogrb.2016.01.001 26802253

[13] Ruder EH Hartman TJ Goldman MB. Impact of oxidative stress on female fertility. Curr Opin Obstet Gynecol. (2009) 21:219–22. 10.1097/GCO.0b013e32832924ba 19469044 PMC2749720

[14] Agarwal A Aponte-Mellado A Premkumar BJ Shaman A Gupta S. The effects of oxidative stress on female reproduction: a review. Reprod Biol Endocrinol. (2012) 10:49. 10.1186/1477-7827-10-49 22748101 PMC3527168

[15] Tamura H Takasaki A Miwa I Taniguchi K Maekawa R Asada H. Oxidative stress impairs oocyte quality and melatonin protects oocytes from free radical damage and improves fertilization rate. J Pineal Res. (2008) 44:280–7. 10.1111/j.1600-079X.2007.00524.x 18339123

[16] Tordjman S Chokron S Delorme R Charrier A Bellissant E Jaafari N. Melatonin: pharmacology, functions and therapeutic benefits. Curr Neuropharmacol. (2017) 15:434–43. 10.2174/1570159X14666161228122115 28503116 PMC5405617

[17] Slominski AT Hardeland R Zmijewski MA Slominski RM Reiter RJ Paus R. Melatonin: a cutaneous perspective on its production, metabolism, and functions. J Invest Dermatol. (2018) 138:490–9. 10.1016/j.jid.2017.10.025 29428440 PMC5828910

[18] Tunon JMJ Trilles PP Molina MG Duvison MH Pastor BM Martin PS A Double-blind, randomized prospective study to evaluate the efficacy of previous therapy with melatonin, myo-inositol, folic acid, and selenium in improving the results of an assisted reproductive treatment. Clin Med Insights Ther. (2017) 9:6 10.1177/1179559X17742902

[19] Jahromi BN Sadeghi S Alipour S Parsanezhad ME Alamdarloo SM. Effect of melatonin on the outcome of assisted reproductive technique cycles in women with diminished ovarian reserve: a double-blinded randomized clinical trial. Iran J Med Sci. (2017) 42:73–8. 28293053 PMC5337768

[20] Pacchiarotti A Carlomagno G Antonini G Pacchiarotti A. Effect of myo-inositol and melatonin versus myo-inositol, in a randomized controlled trial, for improving *in vitro* fertilization of patients with polycystic ovarian syndrome. Gynecol Endocrinol. (2016) 32:69–73. 10.3109/09513590.2015.1101444 26507336

[21] Rizzo P Raffone E Benedetto V. Effect of the treatment with myo-inositol plus folic acid plus melatonin in comparison with a treatment with myo-inositol plus folic acid on oocyte quality and pregnancy outcome in IVF cycles. A prospective, clinical trial. Eur Rev Med Pharmacol Sci. (2010) 14:555–61. 20712264

[22] Mokhtari F Akbari Asbagh F Azmoodeh O Bakhtiyari M Almasi-Hashiani A. Effects of melatonin administration on chemical pregnancy rates of polycystic ovary syndrome patients undergoing intrauterine insemination: a randomized clinical trial. Int J Fertil Steril. (2019) 13:225–9. 10.22074/ijfs.2019.5717 31310077 PMC6642424

[23] Eryilmaz OG Devran A Sarikaya E Aksakal FN Mollamahmutoglu L Cicek N Melatonin improves the oocyte and the embryo in IVF patients with sleep disturbances, but does not improve the sleeping problems. J Assist Reprod Genet. (2011) 28:815–20. 10.1007/s10815-011-9604-y 21748445 PMC3169684

[24] Fernando S Wallace EM Vollenhoven B Lolatgis N Hope N Wong M. Melatonin in assisted reproductive technology: a pilot double-blind randomized placebo-controlled clinical trial. Front Endocrinol. (2018) 9:545. 10.3389/fendo.2018.00545 30283403 PMC6157331

[25] Batioglu AS Sahin U Gurlek B Ozturk N Unsal E. The efficacy of melatonin administration on oocyte quality. Gynecol Endocrinol. (2012) 28:91–3. 10.3109/09513590.2011.589925 21770829

[26] Kim MK Park EA Kim HJ Choi WY Cho JH Lee WS. Does supplementation of *in-vitro* culture medium with melatonin improve IVF outcome in PCOS? Reprod Biomed Online. (2013) 26:22–9. 10.1016/j.rbmo.2012.10.007 23177415

[27] Espino J Macedo M Lozano G Ortiz A Rodriguez C Rodriguez AB. Impact of melatonin supplementation in women with unexplained infertility undergoing fertility treatment. Antioxidants. (2019) 8:E338. 10.3390/antiox8090338 31450726 PMC6769719

[28] Galano A Tan DX Reiter RJ. On the free radical scavenging activities of melatonin's metabolites, AFMK and AMK. J Pineal Res. (2013) 54:245–57. 10.1111/jpi.12010 22998574

[29] Fernandez A Ordonez R Reiter RJ Gonzalez-Gallego J Mauriz JL. Melatonin and endoplasmic reticulum stress: relation to autophagy and apoptosis. J Pineal Res. (2015) 59:292–307. 10.1111/jpi.12264 26201382

[30] Goud PT Goud AP Joshi N Puscheck E Diamond MP Abu-Soud HM. Dynamics of nitric oxide, altered follicular microenvironment, and oocyte quality in women with endometriosis. Fertil Steril. (2014) 102:151–9.e155. 10.1016/j.fertnstert.2014.03.053 24825428

[31] Da Broi MG Navarro PA. Oxidative stress and oocyte quality: ethiopathogenic mechanisms of minimal/mild endometriosis-related infertility. Cell Tissue Res. (2016) 364:1–7. 10.1007/s00441-015-2339-9 26685866

[32] Prasad S Tiwari M Pandey AN Shrivastav TG Chaube SK. Impact of stress on oocyte quality and reproductive outcome. J Biomed Sci. (2016) 23:36. 10.1186/s12929-016-0253-4 27026099 PMC4812655

[33] Zheng M Tong J Li WP Chen ZJ Zhang C. Melatonin concentration in follicular fluid is correlated with antral follicle count (AFC) and *in vitro* fertilization (IVF) outcomes in women undergoing assisted reproductive technology (ART) procedures. Gynecol Endocrinol. (2018) 34:446–50. 10.1080/09513590.2017.1409713 29185361

[34] Brzezinski A. Melatonin in humans. N Engl J Med. (1997) 336:186–95. 10.1056/NEJM199701163360306 8988899

[35] Unfer V Raffone E Rizzo P Buffo S. Effect of a supplementation with myo-inositol plus melatonin on oocyte quality in women who failed to conceive in previous *in vitro* fertilization cycles for poor oocyte quality: a prospective, longitudinal, cohort study. Gynecol Endocrinol. (2011) 27:857–61. 10.3109/09513590.2011.564687 21463230

[36] Seko LM Moroni RM Leitao VM Teixeira DM Nastri CO Martins WP. Melatonin supplementation during controlled ovarian stimulation for women undergoing assisted reproductive technology: systematic review and meta-analysis of randomized controlled trials. Fertil Steril. (2014) 101:154–61.e154. 10.1016/j.fertnstert.2013.09.036 24182414

