# Corrigendum: Melatonin Application in Assisted Reproductive Technology: A Systematic Review and Meta-Analysis of Randomized Trials

**DOI:** 10.3389/fendo.2020.00333

**Published:** 2020-05-19

**Authors:** Kai-Lun Hu, Xiaohang Ye, Siwen Wang, Dan Zhang

**Affiliations:** ^1^Key Laboratory of Reproductive Genetics, Ministry of Education, Department of Reproductive Endocrinology, Women's Hospital, Zhejiang University School of Medicine, Hangzhou, China; ^2^Key Laboratory of Women's Reproductive Health of Zhejiang Province, Hangzhou, China

**Keywords:** melatonin, assisted reproductive technology, randomized trial, *in vitro* fertilization, systematic review and meta-analysis

In the original article, we neglected to include the “National Natural Science Foundation of China” and the “Fundamental Research Funds for the Central Universities” as funders. In addition, we didn't include the grant number “2017YFC1001003” for the National Key Research and Development Program. The corrected Funding statement appears below.

## Funding

“This study was supported by the National Key Research and Development Program of China (2018YFC1005003, 2017YFC1001003), the National Natural Science Foundation of China (No. 81974224, 81771535), the Fundamental Research Funds for the Central Universities, the Natural Science Foundation of Zhejiang Province (No. LZ18H040001), Zhejiang University Scholarship for Outstanding Doctoral Candidates, and Zhejiang University Education Foundation Global Partnership Fund.”

In addition, there were errors in the affiliations of the authors. Affiliation 1 should be “Key Laboratory of Reproductive Genetics, Ministry of Education, Department of Reproductive Endocrinology, Women's Hospital, Zhejiang University School of Medicine, Hangzhou, China.” The following has been added as affiliation 2:

Key Laboratory of Women's Reproductive Health of Zhejiang Province, Hangzhou, China.

The authors apologize for this error and state that this does not change the scientific conclusions of the article in any way. The original article has been updated.

