# Supplementary figures and images for: Melatonin Application in Assisted Reproductive Technology: A Systematic Review and Meta-Analysis of Randomized Trials
Source: Front Endocrinol (Lausanne). 2020 Mar 27;11:160. doi: 10.3389/fendo.2020.00160 (PMC7118201; doi:10.3389/fendo.2020.00160)

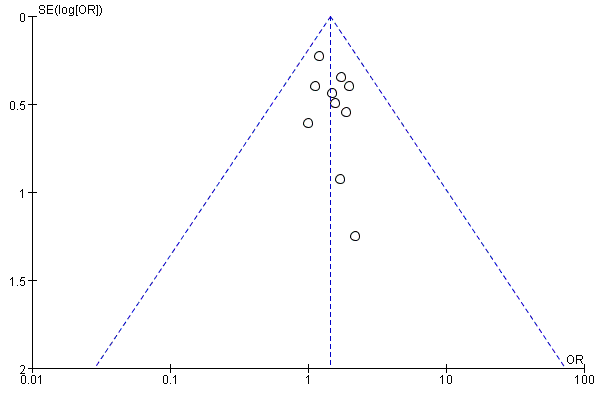

Supplement: Supplemental Figure 1 — Funnel plot of the included studies. [file Image_1.TIF]

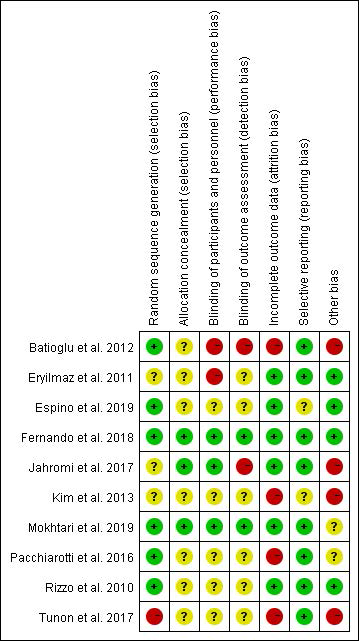

Supplement: Supplemental Figure 2 — Risk of bias summary: review authors' judgments about each risk of bias item for each included study. [file Image_2.TIF]

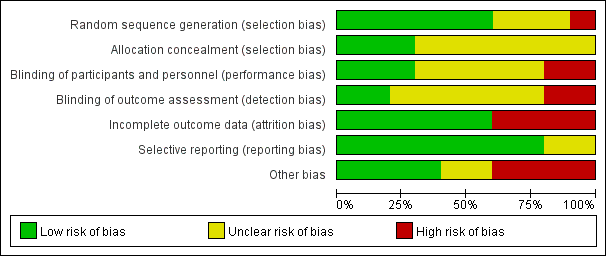

Supplement: Supplemental Figure 3 — Risk of bias graph: review authors' judgments about each risk of bias item presented as percentages across all included studies. [file Image_3.TIF]
