# Supplementary material for: Melatonin Application in Assisted Reproductive Technology: A Systematic Review and Meta-Analysis of Randomized Trials
Source: Front Endocrinol (Lausanne). 2020 Mar 27;11:160. doi: 10.3389/fendo.2020.00160 (PMC7118201; doi:10.3389/fendo.2020.00160)
Supplement: Supplemental Table 1 — Search methods for included studies. [file Data_Sheet_1.docx]

Supplemental table 1. Search methods for included studies.

Pubmed

| #1 | ((((((((((((((((("melatonin"[MeSH Terms]) OR Melatonin[Title/Abstract]) OR ramelteon[Title/Abstract]) OR rozerem[Title/Abstract]) OR circadin[Title/Abstract]) OR agomelatine[Title/Abstract]) OR valdoxan[Title/Abstract]) OR melitor[Title/Abstract]) OR thymanax[Title/Abstract]) OR tasimelteon[Title/Abstract]) OR hetlioz[Title/Abstract]) OR melatonergic[Title/Abstract]) OR melatonin agonist[Title/Abstract]) OR 5 methoxy n acetyltryptamine[Title/Abstract]) OR OR melatonina[Title/Abstract]) OR melovine[Title/Abstract]) OR n acetyl 5 methoxytryptamine[Title/Abstract]) OR slenyto[Title/Abstract] |
| --- | --- |
| #2 | (((((((((((((((((((((((((((((((((((((((((((((((("reproductive techniques, assisted"[MeSH Terms]) OR "fertilization in vitro"[MeSH Terms]) OR insemination, artificial[MeSH Terms]) OR Technique, Assisted Reproductive[Title/Abstract]) OR Techniques, Assisted Reproductive[Title/Abstract]) OR Assisted Reproductive Technics[Title/Abstract]) OR Assisted Reproductive Technic[Title/Abstract]) OR Reproductive Technic, Assisted[Title/Abstract]) OR Reproductive Technics, Assisted[Title/Abstract]) OR Technic, Assisted Reproductive[Title/Abstract]) OR Technics, Assisted Reproductive[Title/Abstract]) OR Assisted Reproductive Techniques[Title/Abstract]) OR Reproductive Technology, Assisted[Title/Abstract]) OR Assisted Reproductive Technologies[Title/Abstract]) OR Assisted Reproductive Technology[Title/Abstract]) OR Reproductive Technologies, Assisted[Title/Abstract]) OR Technologies, Assisted Reproductive[Title/Abstract]) OR Technology, Assisted Reproductive[Title/Abstract]) OR Assisted Reproductive Technique[Title/Abstract]) OR Reproductive Technique, Assisted[Title/Abstract]) OR In Vitro Fertilization[Title/Abstract]) OR In Vitro Fertilizations[Title/Abstract]) OR Test-Tube Fertilization[Title/Abstract]) OR Fertilization, Test-Tube[Title/Abstract]) OR Fertilizations, Test-Tube[Title/Abstract]) OR Test Tube Fertilization[Title/Abstract]) OR Test-Tube Fertilizations[Title/Abstract]) OR Fertilizations in Vitro[Title/Abstract]) OR Test-Tube Babies[Title/Abstract]) OR Babies, Test-Tube[Title/Abstract]) OR Baby, Test-Tube[Title/Abstract]) OR Test Tube Babies[Title/Abstract]) OR Test-Tube Baby[Title/Abstract]) OR IVF[Title/Abstract]) OR Injection, Intracytoplasmic Sperm[Title/Abstract]) OR Injections, Intracytoplasmic Sperm[Title/Abstract]) OR Intracytoplasmic Sperm Injection[Title/Abstract]) OR Sperm Injection, Intracytoplasmic[Title/Abstract]) OR Intracytoplasmic Sperm Injections[Title/Abstract]) OR ICSI[Title/Abstract]) OR Injections, Sperm, Intracytoplasmic[Title/Abstract]) OR Sperm Injections, Intracytoplasmic[Title/Abstract]) OR Eutelegenesis[Title/Abstract]) OR Eutelegeneses[Title/Abstract]) OR Artificial Insemination[Title/Abstract]) OR Artificial Inseminations[Title/Abstract]) OR Inseminations, Artificial[Title/Abstract]) OR intra uterine insemination[Title/Abstract]) OR IUI[Title/Abstract] |
| #3 | ((((((((((((((((((((((((((((((((Live Birth[MeSH Terms]) OR Live Births[Title/Abstract]) OR Live Birth[Title/Abstract]) OR liveborn child[Title/Abstract]) OR liveborn progeny[Title/Abstract]) OR live birth rate[Title/Abstract]) OR Pregnancy Rate[MeSH Terms]) OR Rates, Pregnancy[Title/Abstract]) OR Pregnancy Rates[Title/Abstract]) OR Rate, Pregnancy[Title/Abstract]) OR Pregnancy Rate, Live-Birth[Title/Abstract]) OR Live-Birth Pregnancy Rates[Title/Abstract]) OR Pregnancy Rate, Live Birth[Title/Abstract]) OR Pregnancy Rates, Live-Birth[Title/Abstract]) OR Rate, Live-Birth Pregnancy[Title/Abstract]) OR Rates, Live-Birth Pregnancy[Title/Abstract]) OR Live-Birth Pregnancy Rate[Title/Abstract]) OR Live Birth Pregnancy Rate[Title/Abstract]) OR clinical Pregnancy[Title/Abstract]) OR oocyte quality[Title/Abstract]) OR quality of oocyte[Title/Abstract]) OR egg quality[Title/Abstract]) OR Ovocytes quality[Title/Abstract]) OR Ovocyte quality[Title/Abstract])) OR Ovulation Induction[MeSH Terms]) OR Ovarian Stimulation[Title/Abstract]) OR Ovarian Stimulations[Title/Abstract]) OR Stimulation, Ovarian[Title/Abstract]) OR Stimulations, Ovarian[Title/Abstract]) OR poor responders[Title/Abstract]) OR ovarian response[Title/Abstract] |
| #4 | ((((((((((((((((Clinical Trial[MeSH Terms]) OR Controlled Clinical Trial[MeSH Terms]) OR Randomized Controlled Trial[MeSH Terms]) OR Intervention Study[Title/Abstract]) OR randomized trial[Title/Abstract]) OR trial, clinical[Title/Abstract]) OR clinical trial, controlled[Title/Abstract]) OR controlled clinical comparison[Title/Abstract]) OR controlled clinical drug trial[Title/Abstract]) OR controlled clinical experiment[Title/Abstract]) OR controlled clinical study[Title/Abstract]) OR controlled clinical test[Title/Abstract]) OR controlled trial, randomized[Title/Abstract]) OR randomised controlled study[Title/Abstract]) OR randomised controlled trial[Title/Abstract]) OR randomized controlled study[Title/Abstract]) OR trial, randomized controlled[Title/Abstract] |
|  | #1 AND #2 AND #3 AND #4  7 |

Embase

| 1 | 'melatonin'/exp OR 'melatonin derivative'/exp OR '5 methoxy n acetyltryptamine':ab,ti OR 'circadin':ab,ti OR melatonina:ab,ti OR melovine:ab,ti OR 'n acetyl 5 methoxytryptamine':ab,ti OR ramelteon:ab,ti OR rozerem:ab,ti OR agomelatine:ab,ti OR valdoxan:ab,ti OR melitor:ab,ti OR thymanax:ab,ti OR tasimelteon:ab,ti OR hetlioz:ab,ti OR melatonergic:ab,ti OR 'melatonin agonist':ab,ti OR slenyto:ab,ti |
| --- | --- |
| 2 | 'infertility therapy'/exp OR 'in vitro fertilization'/exp OR 'intrauterine insemination'/exp OR 'assisted reproduction technique':ab,ti OR 'assisted reproduction techniques':ab,ti OR 'assisted reproduction technologies':ab,ti OR 'assisted reproduction technology':ab,ti OR 'assisted reproduction therapy':ab,ti OR 'assisted reproduction treatment;':ab,ti OR 'assisted reproductive technique':ab,ti OR 'assisted reproductive techniques':ab,ti OR 'assisted reproductive technology':ab,ti OR 'assisted reproductive therapy':ab,ti OR 'assisted reproductive treatment':ab,ti OR 'fertility therapy':ab,ti OR 'fertility treatment':ab,ti OR 'reproductive techniques, assisted':ab,ti OR 'extracorporeal fertilization':ab,ti OR 'fertilization in vitro':ab,ti OR 'in vitro fertilisation':ab,ti OR 'ivf (in vitro fertilization)':ab,ti OR 'testtube baby':ab,ti OR 'insemination, intrauterine':ab,ti OR 'uterine insemination':ab,ti |
| 3 | 'live birth'/exp OR 'live birth rate'/exp OR 'clinical pregnancy'/exp OR 'clinical pregnancy rate'/exp OR 'pregnancy'/exp OR 'oocyte quality'/exp OR 'ovarian response'/exp OR 'ovulation induction'/exp OR 'Live Births':ab,ti OR ' liveborn child':ab,ti OR 'liveborn progeny':ab,ti OR 'live birth rate':ab,ti OR 'Pregnancy Rate':ab,ti OR 'Pregnancy Rates':ab,ti OR ' Live-Birth Pregnancy Rates':ab,ti OR ' Live Birth Pregnancy Rates':ab,ti OR 'Live-Birth Pregnancy Rate':ab,ti OR 'Live Birth Pregnancy Rate':ab,ti OR 'egg quality':ti,ab OR 'quality of oocyte':ab,ti OR 'Ovocytes quality':ab,ti OR 'Ovocyte quality':ab,ti OR 'Ovarian Stimulation':ab,ti OR 'Ovarian Stimulations':ab,ti OR 'poor responders':ab,ti OR 'ovarian response':ab,ti |
| 4 | 'randomized controlled trial'/exp OR 'controlled clinical trial'/exp OR 'clinical trial'/exp OR 'Intervention Study':ab,ti OR 'randomized trial':ab,ti OR ' controlled clinical comparison':ab,ti OR 'controlled clinical drug trial':ab,ti OR 'controlled clinical experiment':ab,ti OR 'controlled clinical study':ab,ti OR 'controlled clinical test':ab,ti OR 'randomised controlled study':ab,ti OR 'randomised controlled trial':ab,ti OR ' randomized controlled study':ab,ti |
|  | #1 AND #2 AND #3 AND #4  24 |

Cochranel library

| 1 | MeSH descriptor: [Melatonin] explode all trees OR (melatonin derivative):ti,ab,kw OR (methoxy n acetyltryptamine):ti,ab,kw OR (circadin):ti,ab,kw OR (melatonina):ti,ab,kw OR (melovine):ti,ab,kw OR (n acetyl 5 methoxytryptamine):ti,ab,kw OR (ramelteon):ti,ab,kw OR (rozerem):ti,ab,kw OR (agomelatine):ti,ab,kw OR (valdoxan):ti,ab,kw OR (melitor):ti,ab,kw OR (thymanax):ti,ab,kw OR (tasimelteon):ti,ab,kw OR (hetlioz):ti,ab,kw OR (melatonergic):ti,ab,kw OR (melatonin agonist):ti,ab,kw OR (slenyto):ti,ab,kw |
| --- | --- |
| 2 | MeSH descriptor: [Reproductive Techniques, Assisted] explode all trees OR MeSH descriptor: [Fertilization in Vitro] explode all trees OR MeSH descriptor: [Insemination, Artificial] explode all trees OR (Assisted Reproductive Technics):ti,ab,kw OR (Assisted Reproductive Technic):ti,ab,kw OR (Assisted Reproductive Techniques):ti,ab,kw OR (assisted reproductive technologies):ti,ab,kw OR (Assisted Reproductive Technology):ti,ab,kw OR (Assisted Reproductive Technique):ti,ab,kw OR (In Vitro Fertilization):ti,ab,kw OR (In Vitro Fertilizations):ti,ab,kw OR (Test-Tube Fertilization):ti,ab,kw OR (Test Tube Fertilization):ti,ab,kw OR (Test-Tube Fertilizations):ti,ab,kw OR (Fertilizations in Vitro):ti,ab,kw OR (Test-Tube Babies):ti,ab,kw OR (Test Tube Babies):ti,ab,kw OR (Test-Tube Baby):ti,ab,kw OR (Test Tube Baby):ti,ab,kw OR (Intracytoplasmic Sperm Injection):ti,ab,kw OR (Intracytoplasmic Sperm Injections):ti,ab,kw OR (ivf):ti,ab,kw OR (iui):ti,ab,kw OR (Eutelegenesis):ti,ab,kw OR (Eutelegeneses):ti,ab,kw OR (Artificial Insemination):ti,ab,kw OR (Artificial Inseminations):ti,ab,kw OR (intra uterine insemination):ti,ab,kw |
| 3 | MeSH descriptor: [Live Birth] explode all trees OR MeSH descriptor: [Pregnancy Rate] explode all trees OR MeSH descriptor: [Ovulation Induction] explode all trees OR (Live Births):ti,ab,kw OR (liveborn child):ti,ab,kw OR (liveborn progeny):ti,ab,kw OR (live birth rate):ti,ab,kw OR (Pregnancy Rate):ti,ab,kw OR (Pregnancy Rates):ti,ab,kw OR (Live-Birth Pregnancy Rates):ti,ab,kw OR (Live Birth Pregnancy Rates):ti,ab,kw OR (Live-Birth Pregnancy Rate):ti,ab,kw OR (Live Birth Pregnancy Rate):ti,ab,kw OR (clinical Pregnancy):ti,ab,kw OR (oocyte quality):ti,ab,kw OR (quality of oocyte):ti,ab,kw OR (egg quality):ti,ab,kw OR (Ovocytes quality):ti,ab,kw OR (Ovocyte quality):ti,ab,kw OR (Ovarian Stimulation):ti,ab,kw OR (Ovarian Stimulations):ti,ab,kw OR (poor responders):ti,ab,kw OR (ovarian response):ti,ab,kw |
| 4 | MeSH descriptor: [Controlled Clinical Trial] explode all trees  OR MeSH descriptor: [Randomized Controlled Trial] explode all trees OR MeSH descriptor: [Clinical Trial] explode all trees OR (Intervention Study):ti,ab,kw OR (randomized trial):ti,ab,kw OR (controlled clinical comparison):ti,ab,kw OR (controlled clinical drug trial):ti,ab,kw OR (controlled clinical experiment):ti,ab,kw OR (controlled clinical study'):ti,ab,kw OR (controlled clinical test):ti,ab,kw OR (randomised controlled study):ti,ab,kw OR (randomised controlled trial):ti,ab,kw OR (randomized controlled study):ti,ab,kw |
|  | #1 AND #2 AND #3 AND #4  6 |

| 1 | TOPIC: (Melatonin) OR TOPIC: (ramelteon) OR TOPIC: (rozerem) OR TOPIC: (circadin) OR TOPIC: (agomelatine) OR TOPIC: (valdoxan) OR TOPIC: (melitor) OR TOPIC: (thymanax) OR TOPIC: (tasimelteon) OR TOPIC: (hetlioz) OR TOPIC: (melatonergic) OR TOPIC: (5 methoxy n acetyltryptamine) OR TOPIC: (melatonina) OR TOPIC: (n acetyl 5 methoxytryptamine) OR TOPIC: (slenyto) |
| --- | --- |
|  | TOPIC: (reproductive techniques, assisted) OR TOPIC: (fertilization in vitro) OR TOPIC: (insemination, artificial) OR TOPIC: (Technique, Assisted Reproductive) OR TOPIC: (Assisted Reproductive Technic) OR TOPIC: (Reproductive Technic, Assisted) OR TOPIC: (Technic, Assisted Reproductive) OR TOPIC: (Assisted Reproductive Technique) OR TOPIC: (Reproductive Technology, Assisted) OR TOPIC: (Assisted Reproductive Technologies) OR TOPIC: (Assisted Reproductive Technology) OR TOPIC: (Reproductive Technologies, Assisted) OR TOPIC: (Technologies, Assisted Reproductive) OR TOPIC: (Technology, Assisted Reproductive) OR TOPIC: (Assisted Reproductive Technique) OR TOPIC: (Reproductive Technique, Assisted) OR TOPIC: (In Vitro Fertilization) OR TOPIC: (Test-Tube Fertilization) OR TOPIC: (Fertilization, Test-Tube) OR TOPIC: (Test Tube Fertilization) OR TOPIC: (Fertilizations in Vitro) OR TOPIC: (Test-Tube Babies) OR TOPIC: (Test Tube Babies) OR TOPIC: (Injection, Intracytoplasmic Sperm) OR TOPIC: (Injections, Intracytoplasmic Sperm) OR TOPIC: (Intracytoplasmic Sperm Injection) OR TOPIC: (Sperm Injection, Intracytoplasmic) OR TOPIC: (Intracytoplasmic Sperm Injections) OR TOPIC: (ICSI) OR TOPIC: (Injections, Sperm, Intracytoplasmic) OR TOPIC: (Sperm Injections, Intracytoplasmic) OR TOPIC: (Eutelegenesis) OR TOPIC: (Eutelegeneses) OR TOPIC: (Artificial Insemination) OR TOPIC: (Artificial Inseminations) OR TOPIC: (Inseminations, Artificial) OR TOPIC: (intra uterine insemination) OR TOPIC: (IUI) |
| 3 | TOPIC: (live birth) OR TOPIC: (live birth rate) OR TOPIC: (clinical pregnancy) OR TOPIC: (clinical pregnancy rate) OR TOPIC: (pregnancy) OR TOPIC: (oocyte quality) OR TOPIC: (ovarian response) OR TOPIC: (ovulation induction) OR TOPIC: (Live Births) OR TOPIC: (liveborn child) OR TOPIC: (liveborn progeny) OR TOPIC: (ovarian response) OR TOPIC: (Pregnancy Rate) OR TOPIC: (Pregnancy Rates) OR TOPIC: (Live-Birth Pregnancy Rates) OR TOPIC: (Live Birth Pregnancy Rates) OR TOPIC: (Live-Birth Pregnancy Rate) OR TOPIC: (Live Birth Pregnancy Rate) OR TOPIC: (egg quality) OR TOPIC: (quality of oocyte) OR TOPIC: (Ovocytes quality) OR TOPIC: (Ovocyte quality) OR TOPIC: (Ovarian Stimulation) OR TOPIC: (Ovarian Stimulations) OR TOPIC: (poor responders) |
| 4 | TOPIC: (randomized controlled trial) OR TOPIC: (controlled clinical trial) OR TOPIC: (clinical trial) OR TOPIC: (Intervention Study) OR TOPIC: (randomized trial) OR TOPIC: (controlled clinical comparison) OR TOPIC: (controlled clinical drug trial) OR TOPIC: (controlled clinical experiment) OR TOPIC: (controlled clinical study) OR TOPIC: (controlled clinical test) OR TOPIC: (randomised controlled study) OR TOPIC: (randomised controlled trial) OR TOPIC: (randomized controlled study) |
|  | #1 AND #2 AND #3 AND #4  58 |
